# Intramucosal esophageal dissection caused by gastric tube insertion

**DOI:** 10.1002/ccr3.5996

**Published:** 2022-06-24

**Authors:** Ryo Ichibayashi, Masayuki Watanabe, Mitsuru Honda

**Affiliations:** ^1^ Department of Critical Care Center, Omori Hospital Toho University Medical Center Tokyo Japan

**Keywords:** gastric tube, iatrogenic complications, intramucosal esophageal dissection

## Abstract

We experienced a case of iatrogenic intramucosal esophageal dissection in a patient who had difficulty inserting a gastric tube. CT is useful for diagnosis.

## Abstract 2

It is difficult to insert the gastric tube, and if repeated insertions are attempted, iatrogenic intramucosal esophageal dissection may occur. CT is useful for diagnosis, and imaging should be considered if suspected.

## 1 CLINICAL PICTURE

**FIGURE 1:**
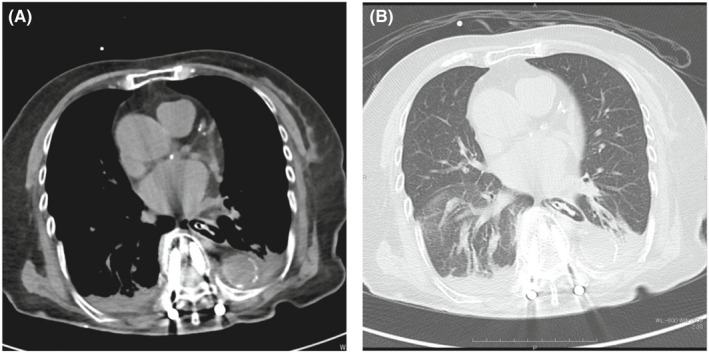
Chest CT

An 82‐year‐old woman was found unconscious. She was diagnosed with subarachnoid hemorrhage. After emergency surgery was performed, the patient was admitted to the intensive care unit. Thereafter, a nasogastric tube insertion was attempted but was found difficult. Thus, a gastric tube insertion using an endoscope was attempted. During endoscope insertion, bleeding in the oral cavity and a hematoma in the right piriform fossa were noted. Chest CT was performed owing to persistent bleeding from the esophageal orifice. Chest CT images via a mediastinal window revealed air circumferentially infiltrating the esophageal wall and extending up to the gastroesophageal junction (Figure 1A). The same site clearly depicted air in the esophageal wall when examined on CT images through a pulmonary window(Figure 1B). Based on these findings, the patient was diagnosed with iatrogenic intramucosal esophageal dissection. Enteral feeding was initiated using a gastric tube within 24 h. On day 3 post‐hospitalization, chest CT revealed the disappearance of intramural emphysema. While CT was performed afterward as appropriate, neither exacerbation of intramucosal esophageal dissection was detected. Intramucosal esophageal dissection is a rare disorder and is classified as traumatic, iatrogenic, and idiopathic.^1^ Endoscopy is a common iatrogenic cause.^2^ In case of difficulty in gastric tube insertion, this disorder may occur. Thus, insertion of the gastric tube should be interrupted, and CT should be performed in suspected cases of this disorder to avoid abscess formation.

## AUTHOR CONTRIBUTIONS

RI wrote and drafted the manuscript. MW and MH helped draft the manuscript. All authors read and approved the final manuscript.

## CONFLICT OF INTEREST

The authors have no conflict of interest to disclose.

## CONSENT

Written informed consent was obtained from the patient to publish this report in accordance with the journal's patient consent policy.

## Data Availability

None.
